# Experiences of liver health related uncertainty and self-reported stress among people who inject drugs living with hepatitis C virus: a qualitative study

**DOI:** 10.1186/s12879-018-3057-1

**Published:** 2018-04-02

**Authors:** Stelliana Goutzamanis, Joseph S. Doyle, Alexander Thompson, Paul Dietze, Margaret Hellard, Peter Higgs, on behalf of the TAP study group

**Affiliations:** 1 Disease Elimination Program, Burnet Institute, 85 Commerical Rd, Melbourne, VIC 3004 Australia; 2 School of Population Health and Preventive Medicine, Monash University, Melbourne, Australia; 3 Department of Infectious Diseases, Alfred Health, Melbourne, Australia; 4 Department of Gastroenterology, St Vincent’s Hospital, Melbourne, Australia; 5 Department of Medicine, University of Melbourne, Melbourne, Australia; 6 Department of Public Health, La Trobe University, Melbourne, Australia

**Keywords:** Hepatitis C virus, People who inject drugs, Stress, Transient elastography, Uncertainty in illness

## Abstract

**Background:**

People who inject drugs (PWID) are most at risk of hepatitis C virus infection in Australia. The introduction of transient elastography (TE) (measuring hepatitis fibrosis) and direct acting antiviral medications will likely alter the experience of living with hepatitis C. We aimed to explore positive and negative influences on wellbeing and stress among PWID with hepatitis C.

**Methods:**

The Treatment and Prevention (TAP) study examines the feasibility of treating hepatitis C mono-infected PWID in community settings. Semi-structured interviews were conducted with 16 purposively recruited TAP participants. Participants were aware of their hepatitis C seropositive status and had received fibrosis assessment (measured by TE) prior to interview. Questions were open-ended, focusing on the impact of health status on wellbeing and self-reported stress. Interviews were voice recorded, transcribed verbatim and thematically analysed, guided by Mishel’s (1988) theory of *Uncertainty in Illness*.

**Results:**

In line with Mishel’s theory of *Uncertainty in Illness* all participants reported hepatitis C-related uncertainty, particularly mis-information or a lack of knowledge surrounding liver health and the meaning of TE results. Those with greater fibrosis experienced an extra layer of prognostic uncertainty. Experiences of uncertainty were a key motivation to seek treatment, which was seen as a way to regain some stability in life. Treatment completion alleviated hepatitis C-related stress, and promoted feelings of empowerment and confidence in addressing other life challenges.

**Conclusion:**

TE scores seemingly provide some certainty. However, when paired with limited knowledge, particularly among people with severe fibrosis, TE may be a source of uncertainty and increased personal stress. This suggests the need for simple education programs and resources on liver health to minimise stress.

## Background

Hepatitis C infection remains a significant public health concern, with an estimated 71 million people chronically infected worldwide [1]. In Australia and other developed countries, the sharing of injecting equipment is the most common source of hepatitis C exposure, placing people who inject drugs (PWID) at greatest risk of infection [2, 3]. Persistent infection may lead to fibrosis, liver disease, hepatocellular carcinoma, liver transplant and extrahepatic manifestations [4, 5]. Fibrosis progression is variable, with approximately 10–20% of individuals with hepatitis C developing cirrhosis within 20–30 years of infection [4, 6, 7]. In Australia, hepatitis C infection is the leading cause of liver transplant, with the burden of hepatitis C liver related disease predicted to triple by 2030 [8].

Recently, the therapeutic landscape of hepatitis C has drastically changed. Non-invasive fibrosis assessment tools, such as transient elastography (TE) (an ultrasound like device which determines liver stiffness by measuring wave velocity [9]) and highly effective interferon-free direct acting antiviral (DAA) medications are now considered standard of care [10, 11]. These advances have made the elimination of hepatitis C as a public health threat a real possibility, particularly in Australia, where DAAs are widely accessible and heavily subsidised [12, 13]. However, such advances may also alter the individual experience of living with hepatitis C.

The qualitative literature has long reported that diagnosis of a chronic illness such as hepatitis C fosters feelings of fear, shock, despair, condemnation, confusion and uncertainty [14–19]. Mishel’s 1981 theory of *Uncertainty in Illness* [20] describes uncertainty as an ‘inability to determine the meaning of, or predict outcomes of illness-related events’ (p 225). This concept of *Uncertainty in Illness* may be manifested through four forms; 1) ambiguity regarding illness state, 2) complexity regarding treatment and system of care, 3) lack of information received or understood, particularly around diagnosis and illness severity and/or 4) unpredictability of disease course and prognosis [20, 21]. Experiences of illness-related uncertainty may jeopardise one’s sense of personal stability, culminating in stress [19, 20]. The term uncertainty is used broadly in this paper to describe the concept of *Uncertainty in Illness.*

Whilst the concept of uncertainty has been the focus of research in populations of people living with Parkinson’s disease [22], Diabetes Mellitus [23], Cancer [24] and Human Immunodeficiency Virus [25, 26], there are few studies exploring uncertainty in people living with hepatitis C [27, 28]. Bailey et al. [27] quantitatively measured uncertainty in people living with hepatitis C who were undergoing ‘watchful and waiting’ protocol, however, did not specify participants’ injecting status. Rhodes, Davis and Judd [28] conducted in-depth interviews with PWID in London and found uncertainty, as a lack of knowledge of hepatitis C prominent, although only 32% of this sample was hepatitis C positive.

The advent of TE to measure the degree of fibrosis means that PWID living with hepatitis C can be more easily made aware of their liver disease progression. Hence, at first blush it seems reasonable to expect that the TE score introduces an aspect of certainty into the lives of those living with hepatitis C. DAA medication provides the prospect of effectively being cured [12], however, for those with pre-existing advanced liver damage, considerable uncertainty about their future health outcomes will remain. Hence, testing and treatment advances may influence the experience of and degree of uncertainty felt by those living with hepatitis C.

This study explores how uncertainty in the context of TE scores and DAA treatment affects self-reported stress in PWID living with hepatitis C. In particular, we focus on how uncertainty in relation to degree of fibrosis and the prospect of receiving treatment is related to stress. Understanding sources of uncertainty in PWID living hepatitis C may aid in developing appropriate resources to reduce hepatitis C related stress.

## Methods

### Participants

Participants were recruited from the Treatment and Prevention (TAP) study, which examines the feasibility of treating hepatitis C mono-infected PWID in a community-based setting [29]. All TAP participants were eligible for qualitative interview provided they had received a valid liver fibrosis assessment and positive (detected) hepatitis C RNA Polymerase Chain Reaction test, at TAP study screening between February 2015 and August 2016. All participants had received a liver stiffness score prior to interviews. Liver stiffness was measured by TE (FibroScan™; Echosense, France). Participants were recruited through a mix of stratified purposive and opportunistic sampling. Participants were grouped as either; ‘high-level fibrosis’ (liver stiffness ≥9.5 Kpa corresponding to METAVIR stage F3/F4) or ‘low-level fibrosis’ (liver stiffness < 9.5 Kpa or F0-F2). Initially a purposive sample of 22 potential participants was selected from TAP, stratified based on age, gender and level of fibrosis (as recorded at trial screening). All 22 participants were contacted via telephone but only two were available for interview (9% response rate). Of these 22 participants the majority had disconnected phone numbers.. Consequently, all eligible TAP participants accessing the trial field sites were sequentially invited to participate and, if they consented, were subsequently interviewed by the first author. Fifteen participants were invited to participate through this method, and 14 were interviewed (93% response rate) including seven participants from the original purposive sample. Through both recruitment methods a total of 16 participants were interviewed. Participants with high-level fibrosis were intentionally ‘over-sampled’ (the proportion of participants with high-level fibrosis in our sample does not reflect the general population) to provide sufficient data to compare experiences between those with high and low-level fibrosis.

### Data collection

Qualitative methodology was employed to gain a more nuanced and in-depth understanding of liver health and wellbeing for PWID living with hepatitis C. Semi-structured interviewing occurred between June and August 2016 across five Melbourne metropolitan sites. Interviews were conducted at community health services, needle and syringe programs or a tertiary hospital.

The interview schedule was informed by the literature on qualitative methodology [30, 31], the research question and discussion between co-authors. Interviews were informal and flexible, using broad prompts such as, ‘what are some things you find stressful at the moment?’ This allowed participants to discuss aspects of life they believed to be impacting on their wellbeing, without being directly asked or feeling obliged to talk about specific factors. Follow-up prompts were on topics such as the physical and emotional effects of hepatitis C and fibrosis and attitudes towards fibrosis and treatment.

Interviews lasted approximately 30 min (range: 17–46 min) and were digitally voice recorded. Participants were reimbursed $AUD40 for their time.

### Data analysis

Participant characteristics collected at TAP screening were identified though pre-assigned unique participant numbers and were utilised to describe the sample. ‘Employed’ included: full-time, part-time or casual employment.

Interviews were transcribed verbatim using Microsoft Word and VLC Media Player (v 2.2.4, VideoLAN Organization, Paris, France). Interviewed participants were assigned unique interview codes and any identifying information was removed from transcripts to ensure anonymity.

Transcripts were read, re-read and then analysed thematically using both inductive and deductive analysis. Deductive codes were based on the four forms of *Uncertainty in Illness* (ambiguity, complexity, lack of information and prognostic unpredictability). Three processes of coding were used; open, axial and thematic [30]. During open coding each line of the transcript was assigned a summative word or phrase. Axial coding focussed on identifying themes from issues that emerged in the open coding stage. This included exploring the physical and emotional impacts of aspects of life that were frequently mentioned. Finally, thematic coding involved understanding what was driving major positive and negative impacts on stress levels and differences between fibrosis groups. The analysis process was conducted iteratively so themes that emerged later in analysis were applied to earlier transcripts. Axial and thematic codes were discussed among authors. Thematic codes were also developed with two independent fieldworkers who regularly interviewed PWID.

All participants provided informed consent and ethics approval was received by the Monash University Human Research Ethics Committee.

## Results

### Sample characteristics

Sixteen interviews were conducted. Participants were aged between 33 and 56 years old (mean: 41 years old ±8.17 years), with most being male (*n* = 11, 69%). Almost all participants were unemployed and had not completed high school. Close to half the sample had high-level fibrosis (*n* = 7, 44%). Four participants had been hepatitis C virus (RNA) positive in the past, but had cleared infection at the time of interview. They were included in analysis for their unique perspective of having recently cleared their infection either spontaneously or through treatment.

### Uncertainty: A key negative impactor on stress

Uncertainty was ubiquitous in the lives of participants we interviewed. Issues that participants reported as stressful consistently appeared to be shrouded in uncertainty or instability, irrespective of the nature of the issue. It was clear that many participants navigated complex aspects of life with trepidation, conceding to feeling generally “lost,” “stuck,” “jumbled up” or “helpless.” These feelings were attributed to a range of factors, including; illness related uncertainty due to their hepatitis C diagnosis, stigma and discrimination, as well as fear of transmission to others. However, themes of stigma, discrimination and fear of transmission have been well described in previous studies [32–35]. Hence, this paper will focus on hepatitis C related uncertainty (ambiguity, lack of information and prognostic unpredictability) and stress, through the scope of TE score, liver health knowledge and DAA treatment.

### Fibrosis related uncertainty: ambiguity and lack of information

Most participants had been diagnosed with hepatitis C many years prior to interview as having “non A, non B” hepatitis or when “Hep C wasn’t even invented”, but had only recently been told their level of fibrosis through the TAP study. Throughout the trial, research nurses experienced in hepatitis C and working with PWID provided participants with information and explanation of TE results. Many participants also received consultation regarding their hepatitis C from other healthcare providers, outside of the TAP study. Despite the consultation with research nurses following their liver assessment; most participants either did not understand or missed key information regarding their TE results, which ignited feelings of stress.

I didn’t know anything about it, [doctors] were like: ‘liver cancer and liver all these things’ and I got really freaked out. (P15, low-level fibrosis)

Approximately half the participants were unclear about the true meaning of TE results, implications of the result and how to manage their fibrosis. Participants were often left feeling frustrated or perplexed when their perceptions of liver health or treatment options did not align with their doctors’, particularly with their liver specialists:

What I understood from [the liver specialist] is that a third is damaged. Well I’m like; ‘can’t you cut a third off?’ Just get rid of that broken bit, it’s simple for me! Then I don’t need to live with the stress. And they’re like; ‘it doesn’t work like that’. That doesn’t make sense to me. (P1, high-level fibrosis)

Confusion over the significance of TE results was also common. TAP participants with a liver stiffness above 9.5Kpa were referred to a clinical site to have their TE test repeated and consult with a specialist clinician. One such participant with high-level fibrosis, who had received a TE result, specialist nurse consultation and referral to a tertiary hospital through TAP immediately prior to our interview, seemed unaware of the meaning of a score indicative of advanced fibrosis:

My liver was 12 something, they had to refer me, anything over 10 she said…even though 12 wasn’t that bad, but because it is over 10 she’s not qualified to do it, so I have to get sent to…what’s that place called? Somewhere to get the big scan. (P4, high-level fibrosis).

There were widespread gaps in knowledge regarding liver health and the TE scores, most of which stemmed from misunderstanding health professionals. This induced a sense of worry and confusion.

### Fibrosis related uncertainty: prognostic uncertainty and unpredictability

All participants demonstrated a lack of knowledge about general liver health irrespective of TE result. However, those with more severe fibrosis expressed a greater degree of worry and stress about how their fibrosis would affect their future than those with low level fibrosis.

Those with low scores generally believed that their degree of fibrosis did not warrant major concern. This appeared to be due to a general lack of symptoms and being told by a health professional that their level of fibrosis was not ‘severe’ or ‘cirrhotic’:

I don’t really put it on my mind, that’s probably why it’s so low. I don’t worry about it. (P13, low-level fibrosis)

[The TE score] was alright, they said it was good. So yeah I don’t think my hep C impacts me that much. (P15, low-level fibrosis)

For this group the certainty of a TE score was useful in preventing worry and stress. In contrast, participants with high-level fibrosis expressed a persistent and palpable worry about their liver health. These participants were often uncertain whether their fibrosis was progressing. This was particularly stressful for those who were not attending TE appointments as regularly as recommended. Some participants felt they no longer knew their TE score and were fearful of becoming aware it had increased:

I’m scared to go to the fibro clinic. My brother in law went…he has cirrhosis of the liver. I’m scared the same thing is going to happen to me. I would like to know my [TE] score but in a way I don't want to know. I'm just lost at the moment. (P6, high-level fibrosis)

The not knowing of the levels, the not knowing how serious it is. I need to know. It’s my life. This is massive! I’ve got grandkids! (P5, high-level fibrosis).

Participants were not only stressed about the state of their livers but seemed unsure of what was expected of them, how to manage their fibrosis, what medical interventions were required and effective and how this would impact their everyday life. The possibility of developing end stage liver disease was a major concern for participants. Multiple participants exhibited a misunderstanding of medical interventions for end-stage liver disease, believing they would require dialysis (an intervention for kidney failure):

I know [my liver] is important cause I might need a new liver or I might need to go on dialysis. I don’t want to end up like that. (P6, high-level fibrosis)

It’s stressful just knowing I’ve damaged my liver. I feel depressed my body is so down, the liver’s not working. Not knowing if I can do anything to fix it is just the worst, because it’s something I didn’t know I got, and now I don’t know how to get rid of it. It’s scary because it’s one of those things that if it collapses you have to be on dialysis or whatever and I’ve seen the dialysis rooms, they have to go and get their blood cleaned, it’s quite scary. (P14, high-level fibrosis)

This degree of prognostic uncertainty, confusion and stress was unique to those interviewed with high-level fibrosis. These participants also frequently reported fatigue-like symptoms such as finding it difficult to exert themselves in the capacity they once could. This restriction on daily living was often difficult to come to terms with, especially as for many it had meant stopping work, giving up hobbies such as bike riding and having to stop consuming alcohol:

Because of the way my liver is, they can’t even give you an answer of what my quality of life will actually be when I’ve finished the Hep C tablets, whether I can go back to work or not. (P1, high-level fibrosis)

### Finding stability through treatment

Hepatitis C was often spoken of as a relatively minor concern in the face of other medical issues, unemployment, financial difficulties, mental illnesses, drug use and dependence, and strained personal relationships. However, with many participants confident they will be cured, hepatitis C was seen as the current and most manageable hurdle.

[Clearing hepatitis C] will help in defeating the bigger problems, because it’s like trying to get up when you’ve got 100 bricks on ya. But then if I took half the bricks off from the Hep C, then now I’ve got a bit more movement and I can start taking the bricks off. (P14, high-level fibrosis)

Not only was treatment seen as achievable but the prospect of becoming ‘cured’ from hepatitis C was viewed as “a lease on life”, a chance to “calm down”, being “comfortable with the kids”, or “normal again”, “one less major problem”, “evening up the playing field”, or clearing “the brain fog.” Underlying these motivations was the desire to ease the stress and uncertainty of living with hepatitis C:

I think it will mean a lot mentally. Just the knowledge that; ‘ahh it’s finally gone.’ I don't think about it but I'm sure I probably do and don't realise it. Once I know it's gone I think it will be a weight off my shoulders. (P8, low-level fibrosis)

Relieving some of the stigma and discrimination participants felt was also a motivator to seek treatment. Participants often described no longer wanting to feel “shameful”, “not normal” or “dirty”. One participant had a history of working in harm reduction services and had observed similar attitudes:

I ask people, ‘why did they feel the need to be treated?’ and a lot of them say because they felt dirty. We aren’t dirty, we just have a virus. (P16, low-level fibrosis)

These motivations were universal across fibrosis levels. Participants who had completed treatment described a sense of relief from uncertainty but also a sense of empowerment:

When I knew I had this sickness there was a fog. To have the doctors say it was gone, it was unreal. I don’t know how to explain it, it felt like butterflies and flowers, I feel alive again, with purpose again and it has motivated me to be better. (P9, high-level fibrosis)

It meant I could live again, I could do anything I wanted and didn't have to worry. (P7, low-level fibrosis)

When participants described their hopes about accessing treatment, it appeared they wanted to minimise or combat feelings of uncertainty. They were seeking stability in their lives. From the narratives of participants who were no longer living with chronic hepatitis C, treatment, either community based (for those with low-level fibrosis) or through tertiary hospitals (for those with high-level fibrosis) had removed many of the hepatitis C related uncertainties that other participants were describing. Not only did treatment reduce the negative feelings associated with hepatitis C and fibrosis, it generated new positive feelings of confidence and an enhanced ability to navigate life’s complexities.

## Discussion

Amidst a rapidly changing therapeutic environment, our study provides insight into the impact of liver health related uncertainty on self-reported stress levels in PWID living with hepatitis C. Our study also revealed motivations for seeking and impact of new DAA treatment in this population. There were three key findings. Firstly, whilst TE is intended to introduce an element of certainty into the lives of those living with hepatitis C, it may be a source of ambiguity, lack of information prognostic uncertainty and ultimately stress. Secondly, liver health uncertainty may be heightened among those with severe fibrosis, as the future appears more unstable, leading to a greater degree of self-reported stress and anxiety. Finally, treatment facilitates a sense of certainty and stability, which allows participants when treated to feel a sense of empowerment, confidence and agency over their life.

In our sample much of the TE related uncertainty was due to limited knowledge. This is consistent with the literature reporting low levels of health literacy and hepatitis C knowledge among PWID living with hepatitis C, which may cause confusion or act as a barrier to treatment [16, 36, 37]. Treloar et al. [38] conducted a self-administered survey with 132 hepatitis C positive clients from Australian opioid substitution therapy clinics. Consistent with our participants’ depictions of fibrosis; the authors noted that levels of knowledge surrounding hepatitis C disease progression were particularly low. Glacken, Kernohan and Coates [16] conducted nine in-depth interviews with people living with hepatitis C. They reported diagnosis related uncertainty as arising from a lack of clinical and prognostic knowledge, which resulted in fear and anxiety. Nevertheless, we found such substantial gaps in knowledge somewhat surprising given participants’ involvement in a hepatitis C treatment trial. For example, numerous participants worried about receiving dialysis if their fibrosis were to progress to liver failure, despite dialysis not being a treatment option for decompensated cirrhosis. However, the broader literature regarding uncertainty in illness suggests that people often receive contradictory information regarding chronic illness [18], do not passively accept expert information but rather reinterpret this knowledge through the scope of lay experiences [39] or may encounter information overload which affects attention and recall [19].

The finding that there was a greater degree of fibrosis related uncertainty and thus self-reported stress among those with more severe fibrosis has not previously been reported, but is not surprising. The combination of awareness of a serious degree of fibrosis, yet little clinical or prognostic knowledge fostered anxiety around life expectancy, disease progression and how fibrosis would impact everyday life. It has not been explored in hepatitis C specifically but others report a positive association between illness severity and uncertainty [19]. Further, quantitative studies suggest that increased fibrosis may be associated with poorer health related quality of life [40, 41]. Bailey et al. [27] tested the Mishel Uncertainty in Illness Scale in 126 untreated people living with chronic hepatitis C. A moderate level of illness uncertainty was seen in this group. Further, the subscales (ambiguity, complexity, inconsistency and unpredictability) were correlated with outcomes such as; depressive symptoms, fatigue, pain and quality of life. However, participants were not distinguished based on disease stage.

There is currently little qualitative published work on the impact of DAA treatment on individuals. Our findings of the overwhelmingly positive psychosocial effects of treatment are also reflected in the treatment stories on numerous hepatitis C blogs and websites [42–44].

Our study was not without limitations. All participants received a TE score prior to interview. However, the time lapse between TE and interview differed between participants, which may have influenced participants’ understanding of their fibrosis. Further, the response rate through the first wave of recruitment was far lower (9% compared to 93%). This is likely due to the first mode of recruitment relying on telephone contact from a telephone number unknown to participants. This is problematic as many PWID do not answer telephone calls from unfamiliar numbers, have limited credit to return calls, may be in contact with the justice system or rehabilitation services and frequently change contact information [45].

## Conclusion

Despite TE seemingly providing people living with hepatitis C some certainty, in our study when paired with limited knowledge, particularly among people with high-level fibrosis it was a source of uncertainty and increased personal stress. These findings suggest that in this setting simple and clear communication from health professionals and education initiatives regarding TE and liver health are required for PWID living with hepatitis C. This may include actively employing the ‘teach-back’ technique. Developing effective and disease stage-specific resources is crucial as it is likely to reduce feelings of stress and anxiety for those with high-level fibrosis and ensure treatment uptake without complacency for those with low-level fibrosis.

## Abbreviations

PWID: People who inject drugs
TAP: Treatment and prevention
TE: Transient elastography

## References

[1] World Health Organisation. Global hepatitis report 2017. Geneva; 2017. Report No.: CC BY-NC-SA 3.0 IGO

[2] Strasser SI, Watson KJ, Lee CS, Coghlan PJ, Desmond PV (1995). Risk factors and predictors of outcome in an Australian cohort with hepatitis C virus infection. Med J Aust.

[3] Dore GJ, Law M, MacDonald M, Kaldor JM (2003). Epidemiology of hepatitis C virus infection in Australia. J Clin Virol.

[4] Chen SL, Morgan TR (2006). The natural history of hepatitis C virus (HCV) infection. Int J Med Sci.

[5] Chin M, Hogan C, Nguyen D (2016). The natural history of hepatitis C viral infection: clinical evaluation and monitoring. Open Med.

[6] Hajarizadeh B, Grebely J, Dore GJ (2013). Epidemiology and natural history of HCV infection. Nat Rev Gastroenterol Hepatol.

[7] Westbrook RH, Dusheiko G (2014). Natural history of hepatitis C. J Hepatol.

[8] Thompson AJ (2016). Australian recommendations for the management of hepatitis C virus infection: a consensus statement. Med J Aust.

[9] Sandrin L, Fourquet B, Hasquenoph J-M, Yon S, Fournier C, Mal F (2003). Transient elastography: a new noninvasive method for assessment of hepatic fibrosis. Ultrasound Med Biol.

[10] European Association for the Study of the Liver (2016). EASL recommendations on treatment of hepatitis C 2016.

[11] Hepatitis C Virus Infection Consensus Statement Working Group (2017). Australian Reccomendations for the management of hepatitis C virus infection: a consensus statement (January 2017).

[12] World Health Organisation (2016). Combating hepatitis B and C to reach elimination by 2030.

[13] Scott N, McBryde ES, Thompson A, Doyle JS, Hellard ME. Treatment scale-up to achieve global HCV incidence and mortality elimination targets: a cost-effectiveness model. Gut. Published Online First: 12 April 2016. 10.1136/gutjnl-2016-311504. 10.1136/gutjnl-2016-311504 27196586

[14] Harris M (2009). Troubling biographical disruption: narratives of unconcern about hepatitis C diagnosis. Sociol Helath Illn.

[15] Crockett B, Gifford SM (2004). “Eyes wide shut”: narratives of women living with hepatitis C in Australia. Women Health.

[16] Glacken M, Kernohan G, Coates V (2001). Diagnosed with hepatitis C: a descriptive exploratory study. Int J Nurs Stud.

[17] Faye B, Irurita V (2003). Balancing perspective: the response to feelings of being condemned with the hepatitis C virus. J Subst Use.

[18] Bury M (1982). Chronic illness as biographical disruption. Sociol Health Illn.

[19] Rice VH (2000). Handbook of stress, coping, and health: implications for nursing research, theory, and practice.

[20] Mishel MH (1981). The measurement of uncertainty in illness. Nurs Res.

[21] Mishel MH (1988). Uncertainty in illness. Image J Nurs Sch.

[22] Sanders-Dewey NEJ, Mullins LL, Chaney JM (2001). Coping style, perceived uncertainty in illness, and distress in individuals with Parkinson's disease and their caregivers. Rehabil Psychol.

[23] Mason C (1985). The production and effects of uncertainty with special reference to diabetes mellitus. Soc Sci Med.

[24] Miller LE (2012). Sources of uncertainty in cancer survivorship. J Cancer Surviv.

[25] Brashers D, Neidig J, Reynolds N, Haas S (1998). Uncertainty in illness across the HIV/AIDS trajectory. J Assoc Nurses AIDS Care.

[26] O’Brien KK, Bayoumi AM, Strike C, Young NL, Davis AM (2008). Exploring disability from the perspective of adults living with HIV/AIDS: development of a conceptual framework. Helath Qual Life Out.

[27] Bailey DE, Landerman L, Barroso J, Bixby P, Mishel MH, Muir AJ (2009). Uncertainty, symptoms and quality of life in persons with chronic hepatitis C undergoing watchful waiting. Psychosomatics.

[28] Rhodes T, Davis M, Judd A (2004). Hepatitis C and its risk management among drug injectors in London: renewing harm reduction in the context of uncertainty. Addiction.

[29] Hellard M, McBryde E, Davis RS, Rolls DA, Higgs P, Aitken C (2015). Hepatitis C transmission and treatment as prevention - the role of the injecting network. Int J Drug Policy.

[30] Liamputtong P, Ezzy D (2005). Qualitative research methods.

[31] Pope C, Mays N (1995). Reaching the parts other methods cannot reach: an introduction to qualitative methods in health and health services research. BMJ.

[32] Conrad S, Garrett LE, Cooksley WG, Dunne MP, MacDonald GA (2006). Living with chronic hepatitis C means ‘you just haven’t got a normal life any more’. Chronic Illn.

[33] Treloar C, Rhodes T (2009). The lived experience of hepatitis C and its treatment among injecting drug users: qualitative synthesis. Qual Health Res.

[34] Lancaster K, Santana L, Madden A, Ritter A (2015). Stigma and subjectivities: examining the textured relationship between lived experience and opinions about drug policy among people who inject drugs. Drugs Educ Prev Policy.

[35] Butt G, Paterson BL, McGuinness LK (2008). Living with the stigma of hepatitis C. West J Nurs Res.

[36] Khaw F-M, Stobbart L, Murtagh MJ (2007). ‘I just keep thinking I haven’t got it because I’m not yellow’: a qualitative study of the factors that influence the uptake of hepatitis C testing by prisoners. BMC Public Health.

[37] Lally MA, Montstream-Quas SA, Tanaka S, Tedeschi SK, Morrow KM (2008). A qualitative study among injection drug using women in Rhode Island: attitudes toward testing, treatment, and vaccination for hepatitis and HIV. AIDS Patient Care ST.

[38] Treloar C, Hull P, Dore GJ, Grebely J (2012). Knowledge and barriers associated with assessment and treatment for hepatitis C virus infection among people who inject drugs. Drug Alcohol Rev.

[39] Royer A, Kronenfeld J (2000). Uncertainty: a key characteristic of chronic illness and a major problem for managed care. Health, illness, and use of care: the impact of social factors.

[40] Björnsson E, Verbaan H, Oksanen A, Frydén A, Johansson J, Friberg S (2009). Health-related quality of life in patients with different stages of liver disease induced by hepatitis C. Scan J Gastroenterol.

[41] Bonkovsky HL, Snow KK, Malet PF, Back-Madruga C, Fontana RJ, Sterling RK (2007). Health-related quality of life in patients with chronic hepatitis C and advanced fibrosis. J Hepatol.

[42] Hepatitis Australia (2017). Hep C treatment stories 2015.

[43] Bager T, Jensen N, Øvrehus A, PB. C, Nielsen D, editors. “I got my life back!”- Patients’ experiences on being cured for hepatitis C: a qualitative study of fatigue and everyday life in a group of danish patients. 5^th^ International Symposium on Hepatitis Care in Substance Users; 2016; Oslo.

[44] Hepatitis Victoria. Personal Stories 2017. 2017 http://www.hepvic.org.au/page/1172/personal-stories. Accessed 4 June 2017.

[45] von Bibra S, Doyle JS, Higgs P, Dietze P, Desmond P, Stoove M (2016). Feasibiliy of recruiting people who inject drugs into a nurse-led model of care trial: the tap study. J Hepatol.

